# Assessment of Ipsilateral Efferent Effects in Human *via* ECochG

**DOI:** 10.3389/fnins.2017.00331

**Published:** 2017-06-08

**Authors:** Eric Verschooten, Elizabeth A. Strickland, Nicolas Verhaert, Philip X. Joris

**Affiliations:** ^1^Laboratory of Auditory Neurophysiology, Department of Neurosciences, University of LeuvenLeuven, Belgium; ^2^Department of Speech, Language, and Hearing Sciences, Purdue UniversityWest Lafayette, IN, United States; ^3^ExpORL Research Group, Department of Neurosciences, University of LeuvenLeuven, Belgium

**Keywords:** precursor, human, CAP, ipsilateral elicitor, efferent, medial olivocochlear system, MOC, ECochG

## Abstract

Development of electrophysiological means to assess the medial olivocochlear (MOC) system in humans is important to further our understanding of the function of that system and for the refinement and validation of psychoacoustical and otoacoustic emission methods which are thought to probe the MOC. Based on measurements in anesthetized animals it has been hypothesized that the MOC-reflex (MOCR) can enhance the response to signals in noise, and several lines of evidence support such a role in humans. A difficulty in these studies is the isolation of efferent effects. Efferent activation can be triggered by acoustic stimulation of the contralateral or ipsilateral ear, but ipsilateral stimulation is thought to be more effective. However, ipsilateral stimulation complicates interpretation of effects since these sounds can affect the perception of other ipsilateral sounds by mechanisms not involving olivocochlear efferents. We assessed the ipsilaterally evoked MOCR in human using a transtympanic procedure to record mass-potentials from the cochlear promontory or the niche of the round window. Averaged compound action potential (CAP) responses to masked probe tones of 4 kHz with and without a precursor (designed to activate the MOCR but not the stapedius reflex) were extracted with a polarity alternating paradigm. The masker was either a simultaneous narrow band noise masker or a short (20-ms) tonal ON- or OFF-frequency forward masker. The subjects were screened for normal hearing (audiogram, tympanogram, threshold stapedius reflex) and psychoacoustically tested for the presence of a precursor effect. We observed a clear reduction of CAP amplitude by the precursor, for different masking conditions. Even without an MOCR, this is expected because the precursor will affect the response to subsequent stimuli via neural adaptation. To determine whether the precursor also activated the efferent system, we measured the CAP over a range of masker levels, with or without precursor, and for different types of masker. The results show CAP reduction consistent with the type of gain reduction caused by the MOCR. These results generally support psychoacoustical paradigms designed to probe the efferent system as indeed activating the MOCR system, but not all observations are consistent with this mechanism.

## Introduction

An important property of the cochlea is the ability to “amplify” the mechanical vibrations at the basilar membrane (Dallos, 2008). This process is under the control of the medial olivocochlear (MOC) system via efferent fibers that innervate the outer hair cells. Activation of these efferents, called the MOC reflex (MOCR), hyperpolarizes the outer hair cells (Fuchs, 2002) and decreases the cochlear gain in anesthetized animals (Buno, 1978; Dolan and Nuttall, 1988; Liberman, 1989; Warren and Liberman, 1989; Kawase and Liberman, 1993; Guinan and Stankovic, 1996).

The role of the MOCR in auditory processing is not well-understood. Various proposals have been made, such as increased speech comprehension in noise (Giraud et al., 1997), protection against loud sounds (Kujawa and Liberman, 1997; Brown et al., 1998), and a possible role in the development of cochlear function (Walsh et al., 1998). Further elucidation of the role of the MOCR requires a combination of behavioral and physiological methods.

In humans, 3 basic approaches have been used to study the MOCR. Measurement of otoacoustic emissions while presenting contralateral sounds allows a rather direct probing of effects on outer hair cells (Guinan, 2006), but a drawback is that such measurements do not address effects on the cochlear neural output. This concern is alleviated by the measurement of acoustically evoked neural mass potentials while presenting contralateral stimuli (Folsom and Owsley, 1987; Kawase and Takasaka, 1995; Chabert et al., 2002; Lichtenhan et al., 2016), but in turn these techniques have other issues such as signal quality, state of arousal, and role of pathology in patients. Finally, a range of psychoacoustical paradigms have been developed to study efferent effects (see below). The challenge with behavioral paradigms is to know whether the effects observed indeed reflect the MOCR or whether they involve other neural pathways or phenomena. By probing cochlear neural potentials as directly as possible, in normal hearing subjects, and applying stimulus paradigms as used in psychoacoustical studies, we aim to tighten the interpretation of behavioral and physiological responses with respect to efferent function.

Although in physiological studies the MOCR may be elicited via direct electrical stimulation of the efferent pathway, the MOCR is more naturally activated by sounds to either ear (Gifford and Guinan, 1987). Use of acoustic stimulation of the contralateral ear to trigger efferent activity is appealing because of its technical and interpretational simplicity. However, anatomical and physiological evidence in cat and guinea pig (Liberman and Brown, 1986; Brown, 1989), indicates that the MOCR is more strongly activated by an ipsilateral elicitor than a contralateral one. While this suggests it is important to study ipsilateral elicitors of efferent activation, such elicitors introduce additional effects, such as cochlear suppression and neural adaptation, which complicate the interpretation of the results.

Under certain circumstances, neural responses to tones in noise may increase in amplitude when the MOCR is elicited. This is known as the anti-masking effect and is thought to reflect a decrease in masking due to a reduction in cochlear gain by the MOCR (Kawase and Liberman, 1993; Kawase et al., 1993). Various psychoacoustical paradigms have been developed to study the effect of the MOCR on masking. For example, in studies of the so-called overshoot or temporal effect (Zwicker, 1965), a precursor sound leads to effects which are qualitatively consistent with the neural anti-masking phenomenon (Strickland, 2001, 2004, 2008). The precursor sound is thought to lead to gain reduction by triggering the MOCR. To tease out the role of gain reduction against other cochlear phenomena (neural adaptation, suppression), psychoacoustic experimenters have developed forward masking paradigms in which masking by a short ON- or OFF-frequency masker is compared with and without a precursor (Roverud and Strickland, 2010). In contrast to the simultaneous masking condition, in forward masking the precursor increases signal threshold. However, the precursor increases signal threshold much more when the masker is well-below the signal frequency than when the masker is at the signal frequency, which would be consistent with a reduction in cochlear gain (Jennings et al., 2009; Jennings and Strickland, 2012; Yasin et al., 2014).

The interpretation of psychoacoustical results in terms of MOCR activity would be strengthened by linking psychoacoustical paradigms more directly with physiological measurements. Here, we attempt to electrophysiologically assess the mechanism by which a precursor affects the detection of a masked probe tone. Our stimulus paradigm is similar to the psychoacoustical studies, but modified to extract the compound action potential (CAP) from mass-potentials near the round window. The experiments were performed in two awake subjects. We first examine the impact of a precursor on a probe tone of 4 kHz and then explore the effect of an additional masker. Finally, we compare the results with predictions from simulations.

## Materials and methods

This study (S56783) was carried out in accordance with the recommendations of good clinical practice (ICH/GCP), Medical Ethics Committee of the University of Leuven with written informed consent from all subjects. All subjects gave written informed consent in accordance with the Declaration of Helsinki. The protocol (ECochG-EF-P-2) was approved by the Medical Ethics Committee of the University of Leuven.

### Subjects

We recruited volunteers between 20 and 30 years of age via an advertisement. Subjects were requested to avoid exposure to loud sounds such as rock concerts in the days preceding the experimental session. The day before or the morning of the experimental session, the subject's hearing was assessed including an inquiry for hearing problems, a pure tone audiogram (thresholds <20 dB nHL, 125 Hz–8 kHz), tympanometry to assess middle ear function, an otomicroscopy by an otolaryngologist, and the determination of the ipsilateral acoustically evoked middle ear reflex threshold for broadband noise and a 1 kHz tone (ZODIAC 901).

The duration of these experimental sessions varied between 1 and 4 h; subjects could end the session at any time. The experiments were conducted in a double-walled soundproofed and electrically shielded booth (Industrial Acoustics Company, Niederkrüchten, Germany). Subjects chose a comfortable reclined position on a bed and were asked to remain still during the recordings. When in the booth, subjects and experimenters were grounded to the booth via an antistatic wrist strap. During the actual experiment, an observer was present with the subject in the booth to monitor the status of the subject and to act as an intermediary with the experimenters outside the booth. Two female subjects participated in the electrophysiological experiments in this study.

### Trans-tympanic procedure

A trans-tympanic procedure was used to record evoked mass responses from the human middle ear (Verschooten et al., 2013, 2015). For every subject, a custom silicone ear mold (Dentsply, Aquasil Ultra XLV regular) was made which contained two casted openings to hold tubes of 2 mm diameter for needle insertion, visualization, acoustic stimulation, and calibration. The complete acoustic system was calibrated *in situ* with a probe-microphone (Etymotic Research, ER-7C) close to the tympanic membrane. The earphone-speaker was connected to one of the openings of the ear mold via a plastic T-piece which also served as access port for a rigid endoscope with camera (R. WOLF, 8654.402 25 degree PANOVIEW; ILO electronic GmbH, XE50-eco X-TFT-USB) to visualize the ear canal and tympanic membrane. During the acoustic calibration all openings were sealed with Audalin acrylic impression compound (Microsonic); a tiny opening in one of the tubes prevented static pressure build-up. Before the needle-electrode was inserted, the tympanic membrane and ear canal were locally anesthetized with Bonain's solution (equal amounts of cocaine hydrochloride, phenol and menthol), which was aspirated after about 30 min. A short sterile plastic tube was inserted in the mold to accommodate the sterile needle-electrode. Ground and reference electrodes were connected to the equipment. The needle-electrode (TECA, sterile monopolar disposable, 75 mm × 26G, 902-DMG75-TP), was inserted and gently placed through the tympanic membrane on the cochlear promontory or in the niche of the round window under visual endoscopic control. To maintain its position and to ensure good electrical contact, the needle-electrode was maintained under slight tension with rubber bands supported by a custom frame, which was positioned over the external ear and fastened around the head with Velcro strips. Subjects usually had a short-lasting and vague sensation of touch during insertion of the electrode. The openings of the tubes were sealed with Audalin and the needle-electrode was connected to the preamplifier. The subject's right ear was studied: there was no experimental manipulation of the other ear. The session was terminated within 4 h or when the subject expressed the desire to stop. At the end of the experiment, the needle electrode and ear mold were removed and an otomicroscopic examination was performed. Subjects were requested to keep the ear dry for 10 days following the recording session. An otolaryngologyst was available during the weeks after the experiment to address any worries or for a second checkup.

### Acoustical stimulation

Stimuli were generated with custom software and a digital sound system (Tucker-Davis Technologies, system 2, sample rate: 125 kHz/channel) consisting of a digital-to-analog converter (PD1), a digitally controlled analog attenuator (PA5), a headphone driver (HB7) and an electromagnetically shielded earphone-speaker (Etymotic Research, ER2, 20 Hz–16 kHz) connected with plastic tubing to the ear mold. The stimuli were compensated for the *in situ* calibration.

### Electrophysiological recording

Auditory evoked potentials were measured using a low noise differential preamplifier (Stanford Research Systems, SR560). All contacts were made on the ipsilateral side to the recording: the signal input was connected to the needle-electrode; the reference input was connected to an earlobe clamp (with conductive gel) and the ground input was connected to a standard disposable surface electrode placed at the mastoid. For safety, the battery-operated preamplifier was galvanically isolated (A-M systems, Analog stimulus isolator Model 2200) from the mains-powered equipment outside the sound booth. Before the signal was recorded (TDT, RX8, ~100 kHz/channel, max. SNR 96 dB), stored and analyzed (MATLAB), the signal was further amplified (DAGAN, BVC-700A) and band pass filtered (30 Hz–30 kHz, cut-off slopes 12 dB/octave). All stimuli and recorded signals were monitored on-line (LeCroy, WaveSurfer 24Xs) during the session.

### Analysis and stimulus paradigm

Human acoustically-evoked neural mass responses are smaller than those recorded in common laboratory animals. To improve the signal-to-noise ratio (SNR) of the response, the uncorrelated background noise was reduced by averaging the responses of many repetitions (*n* = 200). The averaged response was then de-noised (smoothed) with a non-causal low-pass filter using an RLOESS function (MATLAB). The RLOESS is a non-parametric robust local regression function using weighted linear squares and a 2nd degree polynomial model, which assigns lower weight to outliers in the regression (the weights are given by the bisquare function with zero weight for deviations greater than six mean absolute deviations). The span of the filter was chosen such that it corresponded to a low-pass cutoff of ~3 kHz, or ~1 kHz for CAP measurements with low SNR (i.e., heavily masked responses). The magnitude of the CAP was obtained between the first positive and first negative peak (P1-N1).

The recordings in the awake subjects occasionally contained artifacts due to sporadic head movements. These artifacts had a significant impact on the background noise and thus also on the SNR of the CAP. Single responses were selectively removed by measuring the individual contributions to the CAP (Jackknife method), and rejecting those that deviated in order to optimize the SNR. Note that the stimulus level of the precursor was kept below the subject's middle ear reflex threshold (90 dB SPL for subject 1 and 80 dB SPL for subject 2).

**Figure 1:**
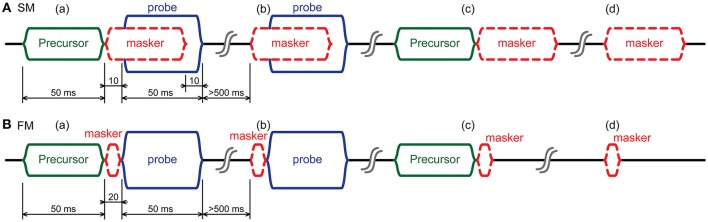
Illustration of the first half presentation of the two stimulus paradigms used in this study. **(A)** paradigm with simultaneous masker and **(B)** with forward masker. Each presentation has 4 segments indicated by letters: **(a)** contains all 3 stimulus components: precursor, masker, and probe; **(b)** similar but without precursor; **(c)** similar but without probe; **(d)** masker only. The probe is always a tone of 4 kHz. The precursor is a broadband noise. The masker can be an ON-frequency (4 kHz) tone; a 2.4 kHz OFF-frequency tone; or a narrowband noise. The second half representation (not shown) is the same as the first, but with all stimuli presented in inverted polarity. A single “condition” consists of the half presentation shown here and the half with opposite polarity. The masker is drawn in dashed lines, indicating the possibility of a condition without masker.

Our stimulus paradigm is designed to assess the mechanism by which a broadband noise precursor affects the detection of a tonal probe of 4 kHz. It is based on psychoacoustical paradigms, but modified to extract the CAP response from mass-potentials near the round window. A first modification is that we employ alternating stimulus polarity to cancel the cochlear microphonic (CM). Second, considerable attention was paid to remove masker artifacts—especially for simultaneous and strong forward maskers—and also to minimize drift between CAPs with different precursor conditions. Drift was expected due to the nature of the recording conditions (movements of awake subjects; varying state of arousal). Figure 1 illustrates the two paradigms, for simultaneous masking (upper) and forward masking (lower). Only the first half presentation, to one stimulus polarity, is shown; the second half is the same, but with opposite polarity. The temporal sequence is such that each paradigm consists of 4 segments. The first segment (a) contains all 3 stimulus components: a probe with a masker and a precursor. The second segment (b) is the same as (a), but without a precursor. The third segment (c) is also the same as segment (a) but without the probe, and the last segment (d) contains only the masker. The duration of the precursor was 50 ms, which has been found to be the optimal length for maximizing gain reduction in psychoacoustic experiments (Roverud and Strickland, 2013). The probe and simultaneous masker were set at the same duration as the precursor. The forward masker was short (20 ms) in order to avoid activation of the MOCR, but long enough to mask the tone. The silent periods between the segments were chosen to be long enough (>500 ms) to allow the MOC-system to recover in between trials.

The probe was always a pure tone of 4 kHz, and the precursor was a Gaussian broadband noise (300–8,000 Hz). The masker was not fixed and changed over experiments and subjects. In the case of forward masking, the masker was either an ON- (4 kHz) or OFF-frequency (2.4 kHz) tone and for simultaneous masking, an OFF-frequency (2.4 kHz) tone or Gaussian narrowband noise (2–6 kHz). The level of the probe was 50, 60, or 70 dB SPL, dependent on subject and masker type. The level of the precursor was fixed to 50 dB SPL and below the subject's threshold of the acoustic reflex. The masker level was the independent variable, but did not exceed 95 dB SPL. Note that measurements with different masker levels were measured in blocks, where the masker level was changed across blocks in arbitrary order.

The rationale for the stimulus design (Figure 1) is as follows. The precursor is designed to activate the MOCR: comparison of segments (a) and (b) will therefore reveal the effect of this activation. Because the MOCR is hypothesized to reduce simultaneous masking, and to increase masking by an OFF-frequency masker more than for an ON-frequency masker, the effect of the precursor is assessed by examining the response to a masker-probe combination. More specifically, we are interested in the response to the probe, which should be reduced by the presence of a masker, and this reduction should change in the presence of a precursor. However, the response to the precursor-masker-probe combination (Figure 1, segment a) contains not only the CAP to the probe tone, but also an off- or on-set and ongoing response to the forward or simultaneous masker. Thus, to isolate the response to the probe, we add conditions in which there is no probe stimulus: a condition with precursor and masker (c) and one without precursor (d). To remove the masker response from (a) and (b), we subtract the responses to (c) and (d), respectively. A disadvantage of such a subtraction procedure is an increase in noise: the mathematical operation to remove the transient response increased the CAP's background noise by 3 dB (summation of two signals with independent background noise signals). For heavily masked responses, where the transient responses to the masker are the largest, we used as compensation the average of segment c and d, which was still satisfactory to suppress the masker's transient response but with less increase in background noise due to the averaging of the two independent background noises inside the compensation signals; the increase in background noise is only 1.6 instead of 3 dB.

We examined the effect of an ipsilateral precursor in simultaneous and forward masking paradigms, which have been used in previous physiological and psychoacoustical studies as described in the Introduction. In simultaneous masking, a release from masking (i.e., an increase in probe response) is expected following a precursor, based on previous physiological studies of the CAP (Kawase and Liberman, 1993) and psychoacoustical studies of overshoot (Zwicker, 1965). In forward masking with an OFF-frequency masker, the precursor will decrease the probe response but not the masker response: so more masking is expected for an OFF-frequency masker than for an on-frequency masker, based on previous psychoacoustical studies (Kawase et al., 2000; Jennings et al., 2009; Jennings and Strickland, 2012).

Forward masking paradigms have the advantage that the different stimulus components do not mutually interact (Figure 1) at the level of the cochlea, and do not induce additional cochlear suppression effects, such as two-tone suppression (e.g., Sachs and Kiang, 1968; Ruggero et al., 1992; van der Heijden and Joris, 2005), which complicate the interpretation of the results.

## Results

A total of five experiments were conducted: 3 in a single session with subject 1, and 2 in a single session with subject 2. The various stimulus conditions used in the two subjects are chronologically listed in Table 1. In all experiments, the masker level was parametrically varied. The first experiment (SM1n) studied masking of a tone in noise using a simultaneous masking paradigm (upper figure in Figure 1), while the other two experiments used OFF- (FM1off) and ON-frequency (FM1on) tonal maskers in a forward masking paradigm (lower figure in Figure 1). In the second session (subject 2), we used only OFF-frequency maskers and compared results with simultaneous (SM2off) and forward (FM2off) maskers. To facilitate comparison between different experiments, CAP responses are expressed as relative values (in %) with respect to the corresponding response without masker.

**Table 1:** Experimental conditions.

**Experiment**	**Precursor 0.3–8 kHz BB-noise**	**Masker**	**Probe 4 kHz tone**
SM1n	50 dB SPL	SM; Quiet, 20, 30, …, 60 dB SPL; 2-6 kHz noise	70 dB SPL
FM1off	50 dB SPL	FM; Quiet, 50, 60, …, 90 dB SPL; 2.4 kHz tone	50 dB SPL
FM1on	50 dB SPL	FM; Quiet, 20, 30, …, 60 dB SPL; 4.0 kHz tone	50 dB SPL
SM2off	50 dB SPL	SM; Quiet, 40, …, 80 dB SPL; 2.4 kHz tone	60 dB SPL
FM2off	50 dB SPL	FM; Quiet, 50, …, 95 dB SPL; 2.4 kHz tone	60 dB SPL

*The names in the first column identify the experiments: the first two characters indicate whether simultaneous masking (SM) or forward masking (FM) was used; the subsequent number indicates the subject; the last characters indicate the stimulus type of the masker (noise or ON- or OFF-frequency masker)*.

### Effect of a precursor without masker

**Figure 2:**
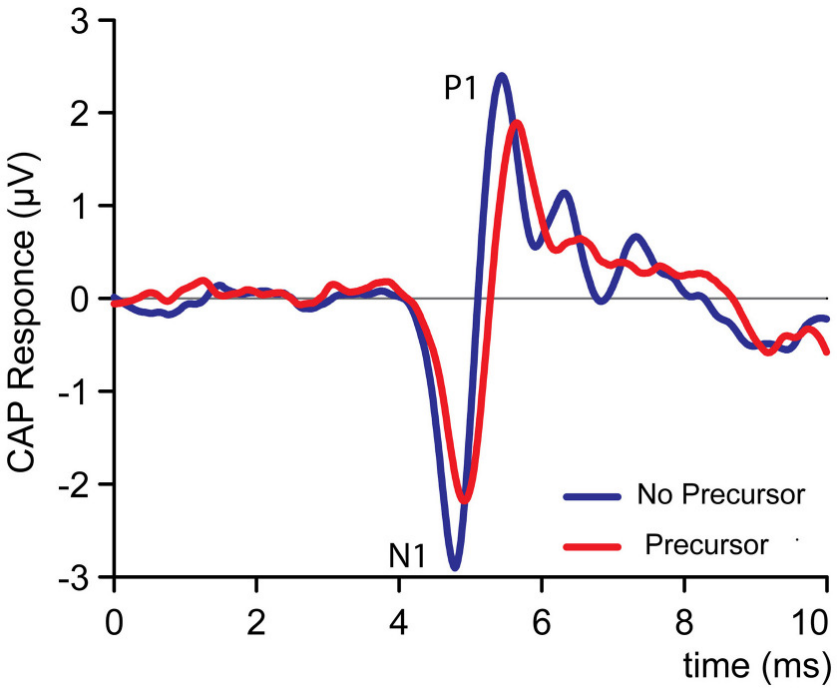
An example of the effect of a broad-band noise precursor of 50 dB SPL on the amplitude and time-course of a human CAP response to a 4 kHz (50 dB SPL) tone, based on >600 averages. The CAP amplitude is measured between P1 and N1. Data is from experiment FM2off.

The precursor is the experimental variable that is intended to activate the MOCR. A difficulty in the study of ipsilateral effects is that the precursor may not only activate efferents but will also have “lingering” or history effects on responses of the same ear to subsequent stimuli even without efferent activation. For convenience, we group such non-efferent history effects (which may contain mechanical, hair cell, synaptic, and neural components) loosely under the term “neural adaptation.” We first examine conditions, present in all experiments, in which there is no effective masker. This gives a first simple assessment of the effect of the precursor on the probe response. Figure 2 shows CAP responses to 4 kHz tones with and without a precursor, from experiment FM2off. Two effects are visible. The CAP amplitude is reduced by the presence of the precursor. Expressing CAP amplitude as the difference in magnitude between the first positive peak P1 and the first negative peak N1, the precursor reduces the CAP magnitude by approximately 20%. Second, the presence of the precursor causes a small delay of 130 μs of N1.

**Figure 3:**
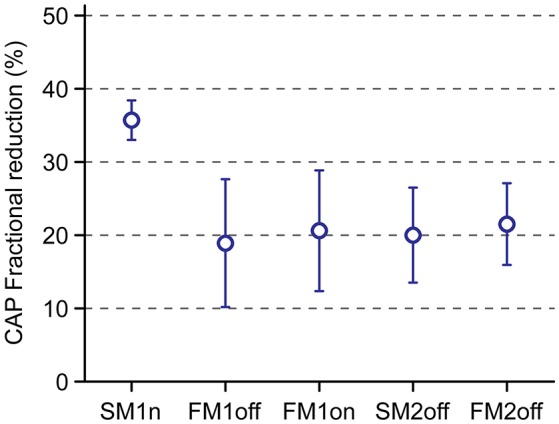
Overview of CAP reduction by a precursor for the different experiments in this study. The error bars indicate propagated SEM obtained with bootstrapping.

Using the same precursor, experiments FM1off, FM1on and SM2off showed a very similar reduction of 20%, as shown in Figure 3. Curiously, the only exception is experiment SM1n, which shows a much greater reduction (35%) compared to the others, as well as smaller variability. Importantly, because Figure 3 is for conditions in which there was no masker, and because the probe frequency and precursor were identical in all experiments, the only stimulus differences were in probe level and in the relative timing between precursor and probe. It appears that the high probe level in experiment SM1n somehow caused a larger effect.

Notwithstanding that the only experiment with somewhat different stimulus conditions gave a deviating result, it is reassuring that the other experiments—where the stimulus conditions were virtually identical—gave rise to very similar effects across experiments and across the two subjects. In the next session, a masker is added to attempt to tease out efferent vs. neural adaptation effects.

### Effect of masker

#### Anti-masking

**Figure 4:**
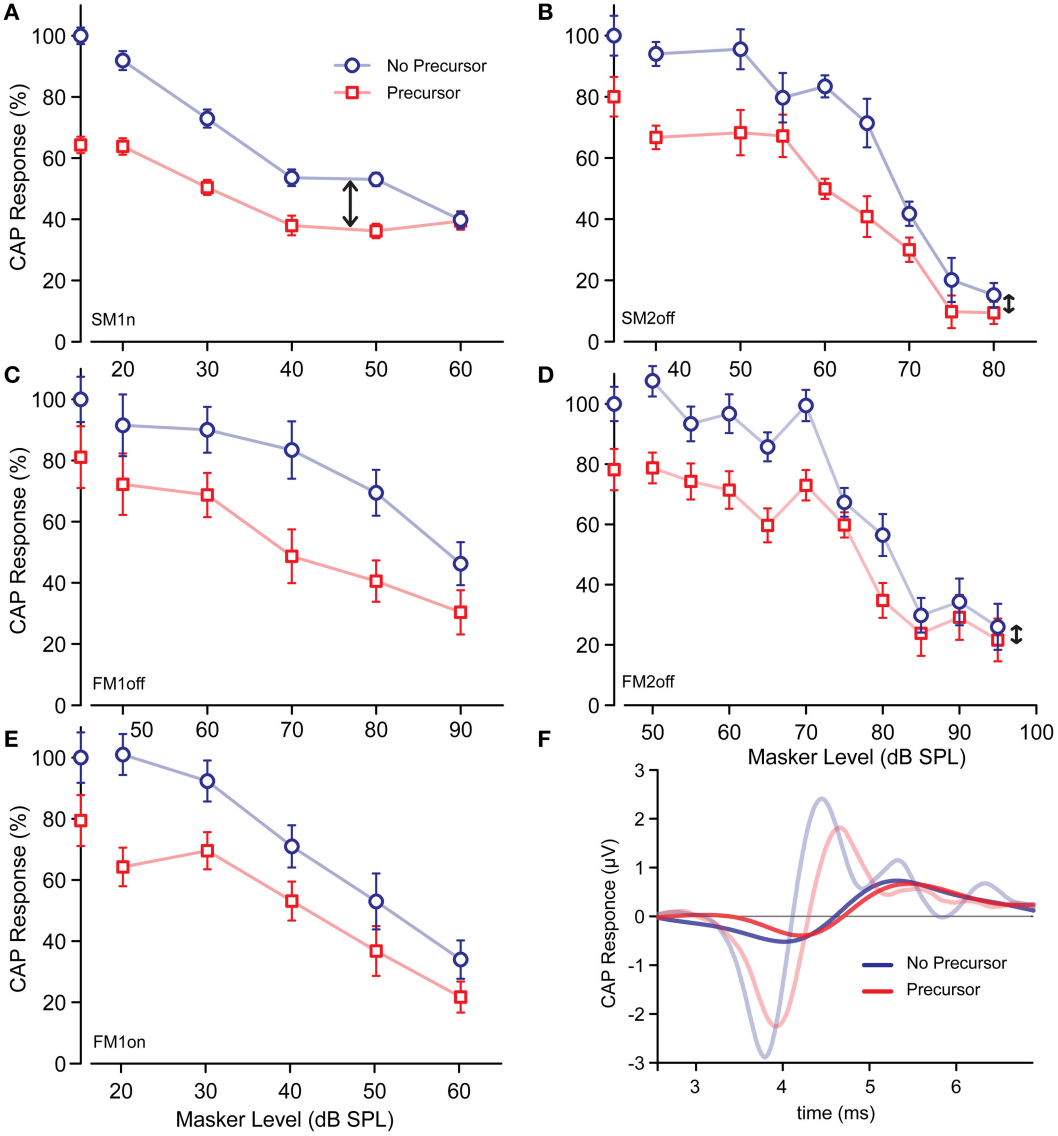
**(A–E)** CAP responses of a 4 kHz masked tone as function of masker levels with (red symbols) and without (blue symbols) BBN precursor, for different experiments. Datapoints on Y-axis are those without masker. **(F)** CAP response at masking saturation of experiment FM2off, with and without precursor. Signals in background are from Figure 2.

Figure 4 shows data for all experiments. We first discuss the overall effect of increasing masker levels, and then the influence of the precursor on that effect, while making abstraction of the different experimental conditions. The blue symbols and lines indicate the probe CAP responses without a precursor. A cursory look at Figures 4A–E shows that, as expected, for all masking configurations an increase in masker level caused a decrease in response to the probe. These curves, which we refer to as standard masking functions, show three regions—not distinct in all experiments. At low masker level there is a region without masking; then a region of active masking where the response declines with masker level; then a region of saturation at high masker levels.

Given that there is masking of the probe response in all experimental conditions, we can look for anti-masking of CAP responses as was shown in anesthetized animals (Kawase and Liberman, 1993), using similar recordings. These investigators found efferent anti-masking effects on CAP responses to tones-in-noise with both forward and simultaneous maskers, which involved both the ipsi- and contra-driven efferent loops. If the noise precursor used here effectively activates the MOCR, the masked response could be larger in the presence of a precursor. This is however never the case (Figures 4A–E): none of the data pairs at any masker level exhibit an increase in response when there is a precursor, so that the red and blue lines and data never cross each other.

The absence of a simple anti-masking effect does not imply that there is no differential MOCR involvement between conditions with or without precursor. The data with a precursor have a similar course (red trendline) as the standard masking curves (blue trendline), but do not asymptote toward the same response values at high masker levels. At low masker levels there is the initial CAP reduction due to the presence of the precursor by itself (Figure 3). This reduction, relative to the condition without precursor, persists at active masker levels. Even at high masker levels, where there is a region of saturation, there remains a constant difference in CAP amplitude between conditions with and without precursor (only exception is at 60 dB for SM1n, Figure 4A, which we consider an outlier). This suggests that the effect of the precursor is not simply one of neural adaptation, because in that case the probe response at high, saturated masker levels would not be affected by the presence or absence of a precursor. We will return to this observation with a quantitative treatment in the final section and figure of Results.

#### Evidence for gain reduction

We now zoom in on a more detailed analysis and comparison of the results of the different experiments and exploit the differences in masker configurations to search for the presence of possible MOCR effects. With tonal ON-frequency maskers, cochlear gain changes due to the MOCR can affect both the probe and masker response. Tonal OFF-frequency maskers, of a frequency lower than the probe, perform masking in the tail of the masker's excitation pattern. Of course, with an OFF-frequency masker, higher masker levels are required to reach masking threshold. OFF-frequency maskers are of interest because they behave linearly with masker level, and, at the tonotopic location of the probe, are believed to be unaffected by the MOCR (Kawase et al., 2000; Cooper and Guinan, 2006). If the precursor indeed triggers the MOCR, this activation will cause a gain reduction for both ON-frequency masker and probe. However, with an OFF-frequency masker a gain reduction due to MOCR activation would only affect the probe and not the masker, effectively making the masker more potent. Thus, the expectation is that, when preceded by a precursor, ON-frequency maskers show a smaller response reduction than OFF-frequency maskers.

**Figure 5:**
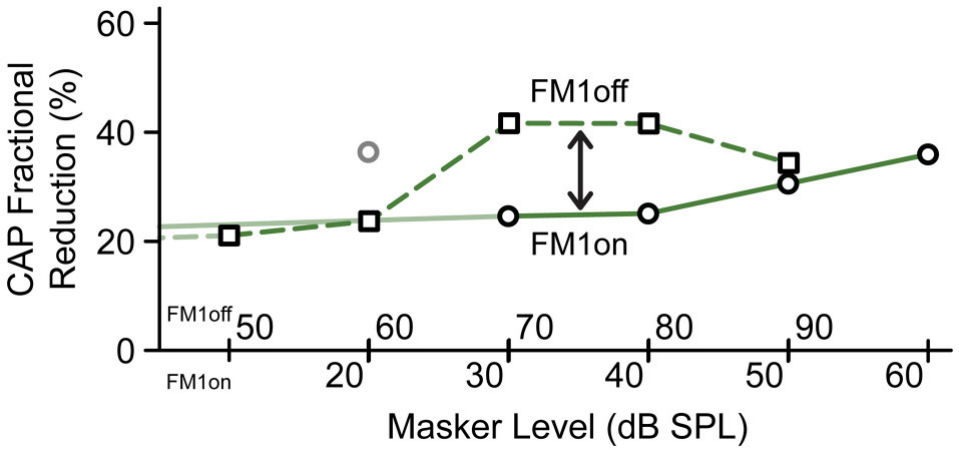
Comparison of CAP response reductions by precursor as function of masker level for ON-and OFF-frequency forward maskers. The masker levels are horizontally offset by 40 dB, according to the midpoint of the masking curves. For each curve, the CAP reduction (in %) is calculated as (CAP response without precursor—CAP responses with precursor)/CAP response without precursor. The datapoint for FM1on at 20 dB is considered an outlier (see also Figure 4E).

**Figure 6:**
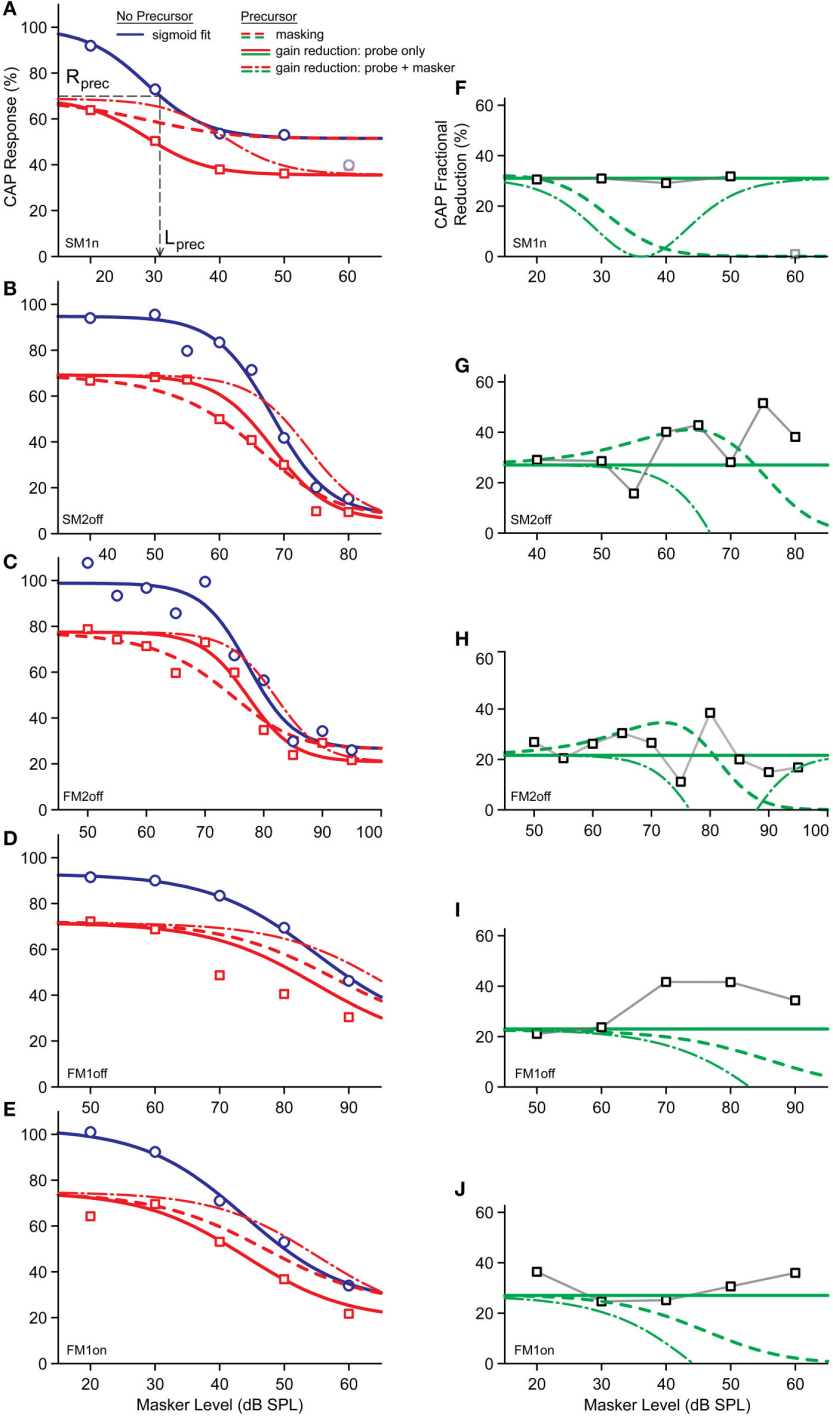
**(A–E)** Predicted masked CAP responses in case the reduction by precursor is from masking (dashed red) or due to the activation of the MOCR (solid red). The data points of the masked responses with precursor are indicated by the red squares; those without precursor by the blue dots. These data points were fit by the blue curve, which was used for the predictions. The dashed gray lines indicated the bias level, Lprec. **(F–J)** Predicted response reductions obtained from the red and blue curves in **(A–E)**. Green dashed curve is for the prediction by masking; the solid green line is the predicted attenuation by the MOCR. The experimental data points are indicated by the black squares.

Figures 4C,E shows the effect of a precursor on the CAP response to a forward masked 4 kHz tone as a function of masker level. Figure 4E shows the results of the ON-frequency masker (experiment FM1on) and Figure 4C that of the OFF-frequency masker (experiment FM1off). Comparison of the two standard masking curves (blue lines, Figures 4C,E), shows, as expected, a rightward shift of ~40 dB for the OFF-frequency masker (value based on sigmoidal fits, explained in Section Predictions from a simple model). This rightward shift is simply due to the fact that it is only through the tail of its excitation pattern that the masker interferes with the probe. When compensated for this level shift, we observe that at active masker levels (i.e., 70, 80 dB SPL for the OFF-frequency masker and 30, 40 dB SPL for the ON-frequency masker) the CAP reduction by precursor is much larger for the OFF- than for ON-frequency maskers. This is illustrated in Figure 5, which shows the CAP reduction induced by the precursor for both experiments. At low masker levels, the same percentage of CAP reduction is observed for ON- and OFF-frequency maskers. At high masker levels, the percentage of CAP reduction is also similar, and presumably reflects gain reduction of the probe response due to the MOCR (see also Figures 6I,J and the final section of RESULTS). However, at masker levels in between, there is indeed a greater reduction by the precursor for the OFF-frequency masker than for the ON-frequency masker, consistent with a reduction in gain by activation of the MOCR (double arrow).

#### Residual reduction at high masker levels

In our discussion of Figure 4 (Section Anti-masking), we remarked that standard masking curves saturate to a certain asymptotic level. At these saturated masker levels, a further decrease in probe response is obtained when a precursor is present. We refer to this as a “residual reduction.” This observation is important because it goes against the reasoning that any contribution by the precursor to neural adaptation can be overwhelmed by a stronger forward masker so that in the limit, at high masker levels, the curves with and without precursor should converge. The residual reduction at saturation suggests an MOCR effect. In the next section, we put this reasoning on a more quantitative footing.

The clearest examples of residual reduction are for Experiments SM2off and FM2off (Figures 4B,D double arrows). CAP responses for FM2off at saturation, with (red) or without (blue) precursor, are illustrated in Figure 4F. For comparison, overlaid in the background, are non-masked responses to these conditions. The masked responses exhibit the same precursor effects as the non-masked responses: a reduction in size and presence of a delay for N1 and P1 (red vs. blue traces). Note also the large delay accompanying the size reduction between non-masked and masked conditions (i.e., the delay between the two red curves and the delay between the two blue curves). Similar residual reductions are present at the highest masker levels in experiments SM1n, FM1off, and FM1on, but for these experiments saturation may not have been reached yet.

Examination of Figure 4 suggests that the size of residual masking by the precursor is related to the size of the remaining response at saturation: the larger the response at saturation (i.e., the larger the blue datapoints at high masker levels), the larger the residual adaptation (i.e., the larger the length of the double arrows). More generally, at all masker levels, the reduction in CAP response between non-precursor and precursor conditions seems to be a constant fraction (between 20 and 30%) across experimental conditions. The observation that this fraction extends to saturated levels of masking suggests that the precursor triggers a constant attenuation of the probe response, consistent with a gain reduction by the MOCR. In Figure 6, we explore this with a phenomenological model and further analysis of the data.

#### Predictions from a simple model

Our model examines the effect of the precursor on the standard masking curve, which is fit by a function. For simplification, only the two most important mechanisms are considered, neural adaptation and reduction in gain. Two important assumptions we make are that the MOCR is modeled by an attenuation due to a reduction in gain; and both mechanisms (MOCR and neural adaptation) are assumed to be independent. We consider 3 situations: Case 1, a response reduction due to neural adaptation by the precursor; Case 2, a gain reduction by the MOCR which affects only the probe but not the masker (cf. OFF-frequency masker); and Case3, the same as Case2 but with an additional “masker release” due to the MOC i.e., an MOC effect on both probe and masker (cf. ON-frequency maskers).

Figures 6A–E shows the trend lines from the model, together with the data points. The blue traces are sigmoidal model fits through the standard masking curves, i.e., data points of the masked responses without a precursor (blue symbols). These fits are obtained with an automated fitting procedure using a modified logistic function (Equation 1).

(1) $$R_{CAP}\left(L_{mask}\right)=\alpha \left(\frac{\left(R_{max}-R_{sat}\right)}{1+\exp\left(k\left(L_{mask}-L_{mid}\right)\right)}+R_{sat}\right)$$

Here, *R*_*CAP*_ is the masked response (in %), *L*_*mid*_ the level of the sigmoid midpoint (dB SPL), *k* determines the steepness of the sigmoid (dB SPL^−1^), *R*_*max*_ is the unmasked CAP response (in %), *R*_*sat*_ is the response at masking saturation (in %), α is an attenuation factor determining the gain reduction by the MOC, and *L*_*mask*_ is the effective masker input level. For the automatic fitting procedure, MATLAB function “*fminsearch*” was used in search for the parameters (i.e., *L*_*mid*_*, R*_*max*_*, R*_*sat*_*, k*) that minimized the RMS-error. Data points were weighted according to their SEM. The data point on the y-axis (Figures 6A–E) is the CAP response without masker (cf. Figure 3): for convenience these are inserted 20 dB below the lowest masker level.

For the standard masker curve, the attenuation (α) was set to 1. In general, the fit to the experimental data is good (Figures 6A–E, blue traces). Note that the data point at the highest masker level in SM1n (Figure 6A) is considered an outlier and was excluded from the dataset. In experiment FM1off (Figure 6D), there were not enough data points in the region of saturation for a proper automated fit, and parameter *R*_*sat*_ was manually chosen based on experiment FM1on.

The red dashed traces in Figures 6A–E represent the predicted trends with precursor for Case 1, thus only including neural adaptation. The same function and fitting parameters were used as for the standard masking curve (blue lines), but with recalculated effective masker input levels (L_m_) to include neural masking by the precursor. Masking by the precursor is simply considered as an additional bias on the existing masking. The bias level was obtained from the standard masking curve as the masker level (*L*_*prec*_) generating a CAP response of the same amplitude as a condition with precursor but without masker (*R*_*prec*_; see Figure 3). *L*_*mask*_ was then recalculated as the square root of the power of *L*_*mask*_ and *L*_*prec*_. This is illustrated by the gray dashed lines in Figure 6A. The *R*_*CAP*_ function so obtained (Figures 6A–E, dashed red line) matched the observed CAP values quite well for SM2off, but not in the other experiments. Clearly, neural adaptation is not adequate to model the effect of the precursor.

The red solid traces (Figures 6A–E) represent the predictions for Case 2, under the assumption that the MOCR induces a gain reduction of the probe only, matching the experimental conditions with OFF-frequency maskers. The same function and fitting parameters were used as for the standard masking curve (blue lines), but with an additional attenuation (α, constant within an experiment) equal to the initial reduction by the precursor, *R*_*prec*_. This prediction clearly outperforms that of Case1 and gives a good fit to the masking data with precursor, except for experiment FM1off, where the predicted masking curve is too far to the right.

Finally, the red dashed-dotted traces (Figures 6A–E) represent the predictions of Case 3, where both masker and probe are affected by a gain reduction caused by the MOCR elicited by the precursor—the situation thought to arise with ON-frequency forward maskers. The same function and fitting parameters were used as for Case 2, but with an additional offset to the masker input level (*L*_*mask*_) to incorporate a gain reduction by the MOC. The size of this additional offset is unknown: we estimate it based on the reduction of the CAP response by the precursor only, as follows. We first determine the maximal slope of the standard masking curve (at *L*_*mid*_ of solid blue line): this slope tells us how to translate a change in CAP response to a change in masker level. We then apply this slope to the reduction of the precursor only (1 – *R*_*prec*_) as follows: offset = (1 – *R*_*prec*_)/absmax(slope of the standard making curve). This offset is the masker threshold shift assuming similar gain reduction as for the probe. Note that—whatever the exact estimate of offset—a reduction in gain of the masker will always shift the masker curve to the right, to higher masker levels (Figures 6A–E, red dashed-dotted lines). A rightward shift actually brings the model prediction further from the observed datapoints than for Case2. Thus, whatever the estimated effect of a gain reduction on the masker, a combined reduction of both masker and probe (Case3) does not give better predictions than gain reduction just of the probe (Case2).

To illustrate the effect of the precursor more directly for these three cases, Figures 6F–J show the percent CAP reductions due to the precursor for the model and the data as a fractional change (% reduction with precursor – % reduction without precursor)/(% reduction without precursor). For Case2, the prediction is simply a horizontal line representing an attenuation or constant gain reduction. For the other two cases, the predicted reductions are strongly dependent on masker level. By and large, the horizontal trend of a constant gain reduction seems to best capture the data.

## Discussion

We assessed the ipsilateral sound-evoked MOCR in humans using CAPs recorded transtympanically in the middle ear using stimulus paradigms similar to previous MOC studies. We measured CAP responses to forward- or simultaneously-masked 4 kHz tones, preceded in some trials by a precursor designed to trigger the MOCR. Some, but not all, of the findings are consistent with MOCR effects as opposed to effects of neural adaptation. First, a noise precursor has a clear reducing effect on unmasked CAP responses (Figures 2, 3). The reduction observed does not seem entirely explainable in terms of neural adaptation. Second, we find residual masking at high masker levels, i.e., while masking saturates at high stimulus levels, a precursor causes further reduction in CAP responses (Figure 4). The behavior of this residual masking is consistent with a gain reduction due to MOCR activation (Figure 6). Third, a comparison between ON- and OFF-frequency maskers showed a clear difference in response reduction by the precursor, consistent with a gain reduction by the MOCR (Figures 4, 5).

### Anti-masking effect

Previous CAP recordings in anesthetized animals show that the MOCR can produce an anti-masking effect, in the sense that CAP responses to a probe tone masked by ipsilateral noise increase in amplitude due to MOCR activation (Kawase and Liberman, 1993). In the latter study, involvement of efferents driven by the ipsilateral ear was detected by sectioning of the olivocochlear bundle which carries efferent fibers from the brainstem to the cochlea. A simple prediction for paradigms as employed in the present study, where the MOCR is triggered by a precursor in the ipsilateral ear, would be that masked CAP responses would increase when preceded by a precursor, relative to the responses without precursor. In the present study, such simple anti-masking effect was not found in any of the stimulus configurations (Figure 4): the datapoints with precursor (red) are always below the datapoints without precursor (blue). However, the absence of such simple anti-masking in the paradigms used in human but not in animals is not very informative and it is misleading to make this comparison. Cutting the olivocochlear bundle allows a clean comparison between responses of a system with and without efferents. The same is not true for the responses with and without precursor: the precursor can affect the responses by mechanisms which are separate from the efferent system. More specifically, the precursor also causes neural adaptation. A more pertinent question therefore is: does the presence of the precursor cause less reduction in masked responses than expected? Answering this question requires a means to disentangle effects of neural adaptation from effects of efferent activation.

### Residual reduction by precursor

Perhaps the most convincing evidence of the presence of an MOCR triggered by the precursor, is the residual reduction of the CAP response at high masker levels. Our reasoning is that exhaustion of neural adaptation manifests itself as saturation of the masking curve at high masker levels (Figure 4). We refer to this as residual reduction, and argue that it is due to a triggering of the MOCR by the precursor. A concern is the reliability of the CAP measurements at high masker levels. Most of the saturated CAPs are quite small and have poor SNR (Figure 4). We took the peak-to-peak amplitude of the CAP to reduce contributions of the summating potential, and also observed that a reduction in amplitude was accompanied by a time delay (Figures 2,4F). Moreover, the presence of residual masking was quite consistent across experiments and across the two subjects. In summary, the data argue that the precursor triggers a process besides neural adaptation which reduces CAP responses.

### Forward masking

One technique used in psychoacoustical experiments to identify an efferent effect is to compare the effectiveness of ON- and OFF-frequency forward maskers. The underlying reasoning is that efferent activity maximally affects basilar membrane vibration near the cochlear location of maximal vibration (active region with gain), and less at more apical or more basal locations with a more linear behavior (Robles and Ruggero, 2001). Thus, while an ON-frequency masker will be rendered less effective by efferent activation, this is less the case for an OFF-frequency masker. We compared the two masker configurations (FM1on and FM1off). Figure 5 shows indeed that the OFF-frequency masker is less affected (remains a stronger masker) by the precursor than for the ON-frequency masker, consistent with a gain reduction for the ON-frequency masker.

Nevertheless, review of the different experiments and quantitative comparisons with predictions from a simple model (Figure 6) reveals a pattern of results that is more complex than anticipated. If the precursor triggers the MOCR so that only the gain to the probe tone (and not to the masker) is affected, a constant CAP reduction is expected across masker levels (horizontal solid line in Figures 6G–I): this is the prediction for an OFF-frequency masker. There is however a tendency in the three experimental conditions with OFF-frequency maskers to display more reduction in fractional change with increasing masker level (i.e., datapoints above the solid horizontal lines in Figures 6G–I). Paradoxically, for the two experiments with ON-frequency maskers, the data very closely do follow the horizontal lines (Figures 6F,J), rather than the prediction for this condition (dash-dotted lines). To put it simply: the results for ON-frequency maskers look as expected for OFF-frequency maskers. The data therefore suggest that in all experiments there is an additional source of reduction of the probe response, which is not adequately modeled by a constant, MOCR-induced, reduction in gain at the probe frequency.

We surmise that a dependency exists between activation of the MOCR and masker level and/or masker type. For example, the shape of the masking curve with precursor might be influenced by the masker level *via* additional activation of the MOCR by the masker itself. In preliminary experiments (not shown) we have observed that efferent activation seems to be biased toward low-frequency stimuli. Although the short masker and slow MOCR activation make it unlikely, there is still a possibility that the presence of a low-frequency, OFF-frequency, masker increasingly contributes to activation of the MOCR with increasing masker level. This would cause additional reduction of the CAP response to the probe (note that the start of the masker always precedes that of the probe, Figure 1, even in the simultaneous masking paradigm). Such increased MOCR activation may explain why there tends to be more reduction of the CAP response with increasing masker level of OFF-frequency maskers (Figures 6G–I) than predicted by the model. With ON-frequency maskers (Figures 6F,J), we modeled the effect of the precursor as a constant attenuation of masker and probe by the MOCR, resulting in the dash-dotted lines, but again the data show more reduction in fractional change than the model. Increased MOCR activation by the increasing masker may be the cause of this additional reduction.

Other factors may add to the complexities of the results, which have more to do with technical aspects of the recorded signals. One issue is that, as masker level increases and CAP amplitude decreases, the nature of the recorded signal may change with a larger reflection of an IHC summating potential. A hint that this may be the case is that the masking curves do not always asymptote to the typically low values seen in animal experiment (Verschooten et al., 2012). Also, there is a possibility that a reflex contraction of the middle ear muscles (MEM) may have affected the recordings, even though the stimuli were below the clinical reflex threshold. We have several reasons to doubt that this was the case. First, muscle activity generates a large signal that is easily detected through the recording electrode, both during online visual and auditory monitoring of the recorded signal, and in the offline analysis (rejection of samples with artifacts). In another study (other subjects), where we used a more intense and longer broadband noise masker, we sometimes observed muscle activity at sound levels which were consistent with the reflex threshold measured with the clinical apparatus. However, in the subjects in this study, such sound-driven MEM artifacts were not observed. Second, another indicator for MEM activation is a significant and systematic decrease in CM amplitude, which is larger for low frequencies but still significantly present for mid and high frequencies (Pang and Guinan, 1997). In our data we did not find a consistent change in CM amplitude over any of the masker levels, including the highest levels at 95 dB SPL. Third, the masker is the stimulus component that reaches the highest levels, and it is present in all stimulus segments (see Figure 1). Considering the short duration of both the masker (20 ms) and its interval to the probe, and the slowness of MEM activation, it is improbable that MEM activation triggered by the masker would differentially affect the responses obtained with and without precursor. To conclude, we think there are sufficient arguments to rule out the possibility that the MEM-reflex rather than the MOCR underlies the effects observed.

### Overshoot effect?

Overshoot is a phenomenon observed in psychoacoustics, which refers to the enhanced detection of a simultaneously-masked pip-tone in the presence of a precursor. The most common hypotheses are that the overshoot is caused by a reduction in gain due to the MOCR (Strickland, 2004; Jennings et al., 2011; Fletcher et al., 2013) or by a reduction in masking due to the adaptive effect of the precursor (Fletcher et al., 2015). As already mentioned (Section Anti-masking effect), none of our electrophysiological experiments revealed an increase in response by the presence of a precursor. We subjected six subjects to a psychoacoustical experiment with a paradigm identical to SM1n, except that the probe tone was shortened to 6 ms. All subjects showed a clear psychoacoustical overshoot, with a consistent masker level increase of ~5 dB (not shown). The absence of an effect in the physiological recordings but not in the psychoacoustical testing does not provide support for the hypothesis that overshoot is caused by a simple gain reduction due to the MOCR, nor by an adaptive effect of the precursor. Rather, in line with conclusions based on psychoacoustical studies (Fletcher et al., 2013, 2015), it is possible that overshoot is a product of central auditory processing operating on peripheral changes that are not detected by our recording methods.

### Effects on CAP waveform

The CAP waveform reflects the summed synchronized discharge of a population of auditory nerve fibers (AN-fibers; Goldstein and Kiang, 1958; Kiang, 1984). Changes in acoustic input or in the processes leading up to the AN responses can affect this summed synchronized population discharge and thereby affect the waveform of the CAP. The most obvious example is the combined change in the waveform's amplitude and latency with input level (Eggermont, 1976; Chabert et al., 2002; Verschooten et al., 2012). In the present study, we focused on effects of the MOCR on CAP amplitude, but, as shown in Figures 2,4F, the precursor also affects latency and shape of the CAP. Particularly the difference in latency at high masker levels, between conditions with and without precursor, suggests that these temporal aspects of the response may help in disambiguating effects of forward masking vs. MOCR (Figure 4F).

**Figure 7:**
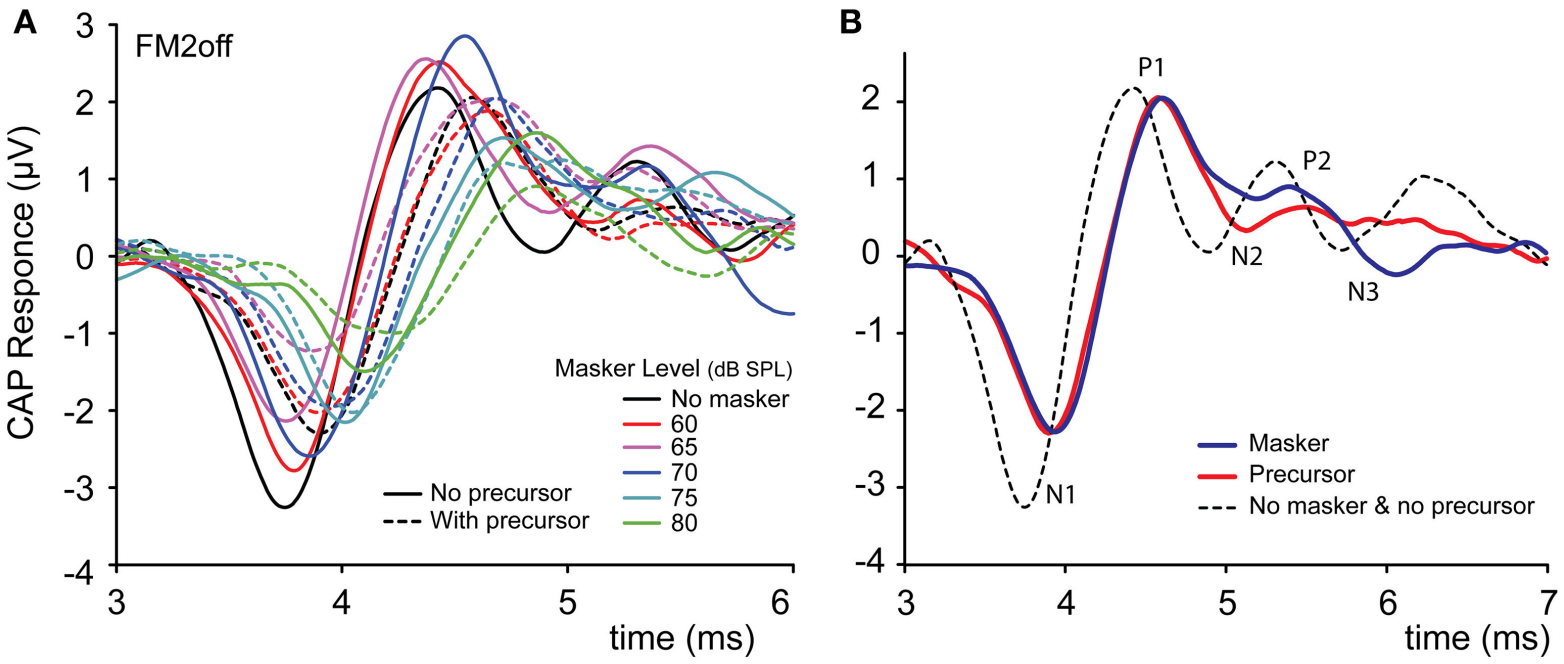
CAP waveforms of experiment FM2off with and without precursor. **(A)** Different masker levels. **(B)** Comparison of CAP waveforms for different conditions: masker-only (blue), precursor-only (red), and without either (dashed). The waveform for the masking-only condition was obtained by interpolating the CAPs for masker levels 70 and 75 dB SPL, such that the CAP magnitude was equal to that of the precursor-only condition.

The processes of gain reduction by the MOCR and of neural adaptation affect AN firing and consequently also the CAP waveform. The overall impact of neural adaptation on the CAP waveform is similar to a reduction in input level (Eggermont, 1979). The solid lines in Figure 7A show indeed that with increasing masker level, CAP amplitudes decrease and latencies increase. Formulating an expectation regarding the effect of an MOCR-induced gain reduction on latency, is more difficult. On the one hand, a reduction in gain is expected to cause a decrease in amplitude and an increase in latency similar to a reduction in input level. On the other hand, several studies report that efferent activation only causes a decrease in CAP amplitude but does not cause a change in latency (e.g., Desmedt et al., 1971; Chabert et al., 2002; Elgueda et al., 2011). In our data, the reduction in CAP amplitude caused by a precursor is accompanied by an increase in latency (Figures 4F,7A: compare solid and dashed lines for a given masker level). While this may at first sight suggest that the CAP reductions caused by the precursor do not reflect activation of the MOCR, but rather neural adaptation, it is important to note that other studies have demonstrated latency effects secondary to efferent activation (e.g., Liberman, 1989; Kawase and Liberman, 1993; Aedo et al., 2015). Possibly, these different outcomes in different studies are related to the type of CAP-evoking stimulus, where studies using clicks show no latency effects but studies using tones do. In any case, it is not clear that examination of the effects on latency allow a better disambiguation of effects of neural adaptation vs. effects of the MOCR.

Neural adaptation and gain reduction by the MOCR operate at different peripheral stages and affect AN-fibers differently. These differences may be reflected not only in amplitude and latency, but also in the precise shape of the CAP waveforms. To illustrate, Figure 7B shows an example of a masker-only (blue line) and precursor-only (red line) responses, that resulted in CAPs identical in amplitude and latency but not in exact waveform shape. The CAP without masker or precursor (dashed line) shows several late waves (e.g., N3,P3): such late features are present in the masker-only condition (blue line) but are more subtle in the precursor-only condition. Possibly, examination of such later features may help to reveal the presence of an MOCR, but a better SNR and availability of additional stimulus conditions would be required for such an effort.

### General considerations

Our expectation was to find an anti-masking effect in CAPs, similar to that observed in anesthetized cat by Kawase and Liberman (1993). Three further points merit consideration. First, especially regarding the comparison of our physiological recordings with psychoacoustical results, it should be remembered that the CAP response only captures a certain aspect of auditory nerve activity (synchronous onset responses). Changes in neural activity that are important for behavioral detection of a probe are not necessarily reflected in the CAP response to this probe. Second, there is a possibility that for some reason (e.g., related to the transtympanic procedure) the MOCR was continuously active during the recording sessions, and that the effect of the presence of the precursor cannot be equated to a simple on or off switching of the MOCR. Third, species differences may be important. In experimental animals, the ipsilateral MOC pathway and reflex is about double in size relative to the contralateral component (Warr, 1992; Guinan, 2011). Anatomical data support the existence of both a lateral and MOC system in humans (Arnesen, 1984; Moore et al., 1999) and, more generally, in primates (Bodian and Gucer, 1980; Thompson and Thompson, 1986), but there is to our knowledge no human anatomical data that addresses anatomical size differences between ipsi- and contralateral MOC systems. Human OAE data suggest that there is little difference between the size of ipsilateral and contralateral MOC reflexes (Guinan, 2006), although more recent data show larger effects for ipsilateral elicitors under certain conditions (Lilaonitkul and Guinan, 2009, 2012).

## Conclusion

It appears that the expected difference between reduction by neural masking and reduction in gain by the MOCR is more subtle and less clear than expected. However, we found several indications of MOC involvement, despite the absence of an anti-masking for tone in noise. Comparison between ON- and OFF-frequency maskers showed a larger reduction by a precursor for OFF than for ON-frequency, consistent with gain reduction. An inconsistency between our model and the data suggests a relationship between the masker level and gain reduction by the MOCR. The most convincing evidence of the presence of a MOCR is the residual response by the precursor at high masker levels.

To conclude, the results in this study show that the response reduction by the precursor is approximately 20–30%. We found that the reduction is fairly independent of masker type, masker level and probe level. These results support psychoacoustical paradigms that are designed to probe the efferent system as indeed activating that system.

## Author contributions

EV, ES, and PJ designed the study; EV and NV performed the measurements; EV analyzed data; EV, ES, and PJ wrote the manuscript.

### Conflict of interest statement

The authors declare that the research was conducted in the absence of any commercial or financial relationships that could be construed as a potential conflict of interest.

## References

[B1] Aedo C. Tapia E. Pavez E. Elgueda D. Delano P. H. Robles L. (2015). Stronger efferent suppression of cochlear neural potentials by contralateral acoustic stimulation in awake than in anesthetized chinchilla. Front. Syst. Neurosci. 9:21. 10.3389/fnsys.2015.00021 25784861 PMC4345911

[B2] Arnesen A. R. (1984). Fibre population of the vestibulocochlear anastomosis in humans. Acta Otolaryngol. 98, 501–518. 10.3109/00016488409107591 6524346

[B3] Bodian D. Gucer G. (1980). Denervation study of synapses of organ of Corti of old world monkeys. J. Comp. Neurol. 192, 785–796. 10.1002/cne.901920411 7419755

[B4] Brown M. C. (1989). Morphology and response properties of single olivocochlear fibers in the guinea pig. Hear. Res. 40, 93–109. 10.1016/0378-5955(89)90103-2 2768087

[B5] Brown M. C. Kujawa S. G. Liberman M. C. (1998). Single olivocochlear neurons in the guinea pig. II. Response plasticity due to noise conditioning. J. Neurophysiol. 79, 3088–3097. 9636110 10.1152/jn.1998.79.6.3088

[B6] Buno W. Jr. (1978). Auditory nerve fiber activity influenced by contralateral ear sound stimulation. Exp. Neurol. 59, 62–74. 10.1016/0014-4886(78)90201-7 627269

[B7] Chabert R. Magnan J. Lallemant J. G. Uziel A. Puel J. L. (2002). Contralateral sound stimulation suppresses the compound action potential from the auditory nerve in humans. Otol. Neurotol. 23, 784–788. 10.1097/00129492-200209000-00029 12218635

[B8] Cooper N. P. Guinan J. J. Jr. (2006). Efferent-mediated control of basilar membrane motion. J. Physiol. 576, 49–54. 10.1113/jphysiol.2006.114991 16901947 PMC1995651

[B9] Dallos P. (2008). Cochlear amplification, outer hair cells and prestin. Curr. Opin. Neurobiol. 18, 370–376. 10.1016/j.conb.2008.08.016 18809494 PMC2630119

[B10] Desmedt J. E. La Grutta V. La Grutta G. (1971). Contrasting effects of centrifugal olivo-cochlear inhibition and of middle ear muscle contraction on the response characteristics of the cat's auditory nerve. Brain Res. 30, 375–384. 10.1016/0006-8993(71)90087-4 5099176

[B11] Dolan D. F. Nuttall A. L. (1988). Masked cochlear whole-nerve response intensity functions altered by electrical stimulation of the crossed olivocochlear bundle. J. Acoust. Soc. Am. 83, 1081–1086. 10.1121/1.396052 3356813

[B12] Eggermont J. J. (1976). Electrocochleography, in Handbook of Sensory Physiology, Vol. 5, Part 3, eds Keidel W. D. Neff W. D. (New York, NY: Springer-Verlag), 626–705.

[B13] Eggermont J. J. (1979). Compound action potentials: tuning curves and delay times. Scand. Audiol. Suppl. 129–139. 294676

[B14] Elgueda D. Delano P. H. Robles L. (2011). Effects of electrical stimulation of olivocochlear fibers in cochlear potentials in the chinchilla. J. Assoc. Res. Otolaryngol. 12, 317–327. 10.1007/s10162-011-0260-9 21365333 PMC3085692

[B15] Fletcher M. de Boer J. Krumbholz K. (2013). Is overshoot caused by an efferent reduction in cochlear gain? Adv. Exp. Med. Biol. 787, 65–72. 10.1007/978-1-4614-1590-9_8 23716210

[B16] Fletcher M. de Boer J. Krumbholz K. (2015). Is off-frequency overshoot caused by adaptation of suppression? J. Assoc. Res. Otolaryngol. 16, 241–253. 10.1007/s10162-014-0498-0 25468405 PMC4368652

[B17] Folsom R. C. Owsley R. M. (1987). N1 action potentials in humans. Influence of simultaneous contralateral stimulation. Acta Otolaryngol. 103, 262–265. 10.3109/00016488709107281 21449650

[B18] Fuchs P. (2002). The synaptic physiology of cochlear hair cells. Audiol. Neurootol. 7, 40–44. 10.1159/000046862 11914525

[B19] Gifford M. L. Guinan J. J. Jr. (1987). Effects of electrical stimulation of medial olivocochlear neurons on ipsilateral and contralateral cochlear responses. Hear. Res. 29, 179–194. 10.1016/0378-5955(87)90166-3 3624082

[B20] Giraud A. L. Garnier S. Micheyl C. Lina G. Chays A. Chery-Croze S. (1997). Auditory efferents involved in speech-in-noise intelligibility. Neuroreport 8, 1779–1783. 10.1097/00001756-199705060-00042 9189932

[B21] Goldstein M. H. Kiang N. Y. S. (1958). Synchrony of neural activity in electric responses evoked by transient acoustic stimuli. J. Acoust. Soc. Am. 30, 107–114. 10.1121/1.1909497

[B22] Guinan J. J. Jr. Stankovic K. M. (1996). Medial efferent inhibition produces the largest equivalent attenuations at moderate to high sound levels in cat auditory-nerve fibers. J. Acoust. Soc. Am. 100, 1680–1690. 10.1121/1.416066 8817894

[B23] Guinan J. J. Jr. (2006). Olivocochlear efferents, anatomy, physiology, function, and the measurement of efferent effects in humans. Ear Hear. 27, 589–607. 10.1097/01.aud.0000240507.83072.e7 17086072

[B24] Guinan J. J. Jr. (2011). Physiology of the medial and lateral olivocochlear systems, in Auditory and Vestibular Efferents, eds Ryugo D. K. Fay R. R. Popper A. N. (New York, NY: Springer Science), 39–81.

[B25] Jennings S. G. Strickland E. A. (2012). Evaluating the effects of olivocochlear feedback on psychophysical measures of frequency selectivity. J. Acoust. Soc. Am. 132, 2483–2496. 10.1121/1.4742723 23039443 PMC3477188

[B26] Jennings S. G. Heinz M. G. Strickland E. A. (2011). Evaluating adaptation and olivocochlear efferent feedback as potential explanations of psychophysical overshoot. J. Assoc. Res. Otolaryngol. 12, 345–360. 10.1007/s10162-011-0256-5 21267622 PMC3085687

[B27] Jennings S. G. Strickland E. A. Heinz M. G. (2009). Precursor effects on behavioral estimates of frequency selectivity and gain in forward masking. J. Acoust. Soc. Am. 125, 2172–2181. 10.1121/1.3081383 19354393 PMC2736734

[B28] Kawase T. Liberman M. C. (1993). Antimasking effects of the olivocochlear reflex. I. Enhancement of compound action potentials to masked tones. J. Neurophysiol. 70, 2519–2532. 8120596 10.1152/jn.1993.70.6.2519

[B29] Kawase T. Takasaka T. (1995). The effects of contralateral noise on masked compound action potential in humans. Hear Res. 91, 1–6. 10.1016/0378-5955(95)00145-X 8647711

[B30] Kawase T. Delgutte B. Liberman M. C. (1993). Antimasking effects of the olivocochlear reflex. II. Enhancement of auditory-nerve response to masked tones. J. Neurophysiol. 70, 2533–2549. 8120597 10.1152/jn.1993.70.6.2533

[B31] Kawase T. Ogura M. Hidaka H. Sasaki N. Suzuki Y. Takasaka T. (2000). Effects of contralateral noise on measurement of the psychophysical tuning curve. Hear. Res. 142, 63–70. 10.1016/S0378-5955(00)00010-1 10748329

[B32] Kiang N. Y. S. (1984). Peripheral neural processing of auditory information, in Handbook of Physiology, Vol. 3, eds Brookhart J. M. Mountcastle V. B. (Bethesda, MD: American Physiological Society), 639–674.

[B33] Kujawa S. G. Liberman M. C. (1997). Conditioning-related protection from acoustic injury: effects of chronic deefferentation and sham surgery. J. Neurophysiol. 78, 3095–3106. 9405529 10.1152/jn.1997.78.6.3095

[B34] Liberman M. C. (1989). Rapid assessment of sound-evoked olivocochlear feedback: suppression of compound action potentials by contralateral sound. Hear. Res. 37, 47–122. 10.1016/0378-5955(89)90127-5 2708159

[B35] Liberman M. C. Brown M. C. (1986). Physiology and anatomy of single olivocochlear neurons in the cat. Hear. Res. 24, 17–36. 10.1016/0378-5955(86)90003-1 3759672

[B36] Lichtenhan J. T. Wilson U. S. Hancock K. E. Guinan J. J. Jr. (2016). Medial olivocochlear efferent reflex inhibition of human cochlear nerve responses. Hear. Res. 333, 216–224. 10.1016/j.heares.2015.09.001 26364824 PMC4788580

[B37] Lilaonitkul W. Guinan J. J. Jr. (2009). Human medial olivocochlear reflex, effects as functions of contralateral, ipsilateral, and bilateral elicitor bandwidths. J. Assoc. Res. Otolaryngol. 10, 459–470. 10.1007/s10162-009-0163-1 19263165 PMC3084380

[B38] Lilaonitkul W. Guinan J. J. Jr. (2012). Frequency tuning of medial-olivocochlear-efferent acoustic reflexes in humans as functions of probe frequency. J. Neurophysiol. 107, 1598–1611. 10.1152/jn.00549.2011 22190630 PMC3311677

[B39] Moore J. K. Simmons D. D. Guan Y. (1999). The human olivocochlear system: organization and development. Audiol. Neurootol. 4, 311–325. 10.1159/000013855 10516391

[B40] Pang X. D. Guinan J. J. Jr. (1997). Effects of stapedius-muscle contractions on the masking of auditory-nerve responses. J. Acoust. Soc. Am. 102, 3576–3586. 10.1121/1.420399 9407651

[B41] Robles L. Ruggero M. A. (2001). Mechanics of the mammalian cochlea. Physiol. Rev. 81, 1305–1352. 11427697 10.1152/physrev.2001.81.3.1305 PMC3590856

[B42] Roverud E. M. Strickland E. A. (2013). Modeling effects of precursor duration on behavioral estimates of cochlear gain. Adv. Exp. Med. Biol. 787, 55–63. 10.1007/978-1-4614-1590-9_7 23716209

[B43] Roverud E. Strickland E. A. (2010). The time course of cochlear gain reduction measured using a more efficient psychophysical technique. J. Acoust. Soc. Am. 128, 1203–1214. 10.1121/1.3473695 20815456 PMC2945748

[B44] Ruggero M. A. Robles L. Rich N. C. (1992). Two-tone suppression in the basilar membrane of the cochlea: mechanical basis of auditory-nerve rate suppression. J. Neurophysiol. 68, 1087–1099. 1432070 10.1152/jn.1992.68.4.1087

[B45] Sachs M. B. Kiang N. Y. (1968). Two-tone inhibition in auditory-nerve fibers. J. Acoust. Soc. Am. 43, 1120–1128. 10.1121/1.1910947 5648103

[B46] Strickland E. A. (2001). The relationship between frequency selectivity and overshoot. J. Acoust. Soc. Am. 109, 2062–2073. 10.1121/1.1357811 11386558

[B47] Strickland E. A. (2004). The temporal effect with notched-noise maskers: analysis in terms of input-output functions. J. Acoust. Soc. Am. 115, 2234–2245. 10.1121/1.1691036 15139634

[B48] Strickland E. A. (2008). The relationship between precursor level and the temporal effect. J. Acoust. Soc. Am. 123, 946–954. 10.1121/1.2821977 18247897 PMC2637526

[B49] Thompson G. C. Thompson A. M. (1986). Olivocochlear neurons in the squirrel monkey brainstem. J. Comp. Neurol. 254, 246–258. 10.1002/cne.902540208 3540042

[B50] van der Heijden M. Joris P. X. (2005). The speed of auditory low-side suppression. J. Neurophysiol. 93, 201–209. 10.1152/jn.00554.2004 15342714

[B51] Verschooten E. Desloovere C. Joris P. (2015). Human neural tuning estimated from compound action potentials in normal hearing human volunteersm, Mechanics of Hearing: Protein to Perception, eds Karavitaki K. D. Corey D. P. (Melville, NY: American Institute of Physics), 070001.

[B52] Verschooten E. (2013). Assessment of Fundamental Cochlear Limits of Frequency Resolution and Phase-Locking in Humans and Animal Models. Ph.D. thesis, KU Leuven.

[B53] Verschooten E. Robles L. Kovacic D. Joris P. X. (2012). Auditory nerve frequency tuning measured with forward-masked compound action potentials. J. Assoc. Res. Otolaryngol. 13, 799–817. 10.1007/s10162-012-0346-z 22948475 PMC3505591

[B54] Walsh E. J. McGee J. McFadden S. L. Liberman M. C. (1998). Long-term effects of sectioning the olivocochlear bundle in neonatal cats. J. Neurosci. 18, 3859–3869. 9570815 10.1523/JNEUROSCI.18-10-03859.1998 PMC6793155

[B55] Warr W. (1992). Organization of olivocochlear efferent systems in mammals, in Mammalian Auditory Pathway: Neuroanatomy, eds Webster D. B. Popper A. N. Fay R. R. (New York, NY: Springer-Verlag), 410–448.

[B56] Warren E. H. Liberman M. C. (1989). Effects of contralateral sound on auditory-nerve responses. I. Contributions of cochlear efferents. Hear Res. 37, 89–104. 2914811 10.1016/0378-5955(89)90032-4

[B57] Yasin I. Drga V. Plack C. J. (2014). Effect of human auditory efferent feedback on cochlear gain and compression. J. Neurosci. 34, 15319–15326. 10.1523/JNEUROSCI.1043-14.2014 25392499 PMC4228134

[B58] Zwicker E. (1965). Temporal effects in simultaneous masking and loudness. J. Acoust. Soc. Am. 38, 132–141. 10.1121/1.1909588 14347604

